# Aorto‑esophageal Fistula following Botox Injection

**DOI:** 10.5334/jbsr.3868

**Published:** 2025-03-25

**Authors:** Thomas Saliba, Denis Tack

**Affiliations:** 1 Centre hospitalier universitaire vaudois (CHUV), Lausanne, Switzerland; 2 Hopital D’Ath (EpiCURA), Ath, Belgium

**Keywords:** Aorta, esophagus, aorto‑esophageal fistula, complication, botox

## Abstract

Aorto‑esophageal fistulas (AEF) are rare, often fatal connections between the thoracic aorta and esophagus, arising from aortic disease, esophageal conditions, or iatrogenic causes.

*Case:* A 76‑year‑old woman, treated for esophageal nutcracker syndrome with endoscopic injection of botox, developed chest pain and esophageal hemorrhage. Computed tomography (CT) confirmed an AEF from an aortic pseudoaneurysm. She succumbed to circulatory collapse before treatment.

*Discussion:* Endoscopic botox injections are a rare cause of AEF. The diagnosis relies on imaging and endoscopy, with surgery often required.

*Teaching point:* Aorto‑esophageal fistulas are rare, life‑threatening complications, particularly after iatrogenic procedures. Early diagnosis is crucial, but prognosis remains poor.

## Introduction

Aorto‑esophageal fistulas (AEF), defined as a connection between the thoracic aorta and the esophagus, are extremely rare and often fatal [1]. These can arise after complications of aortic or esophageal diseases, such as aortic aneurysms, esophageal tumors, foreign body perforations (referred to as primary AEF), or postoperative complications (known as secondary AEF) [1, 2]. AEF is life‑threatening, with a poor prognosis if not immediately treated [1, 3].

## Case Report

**Figure 1:**
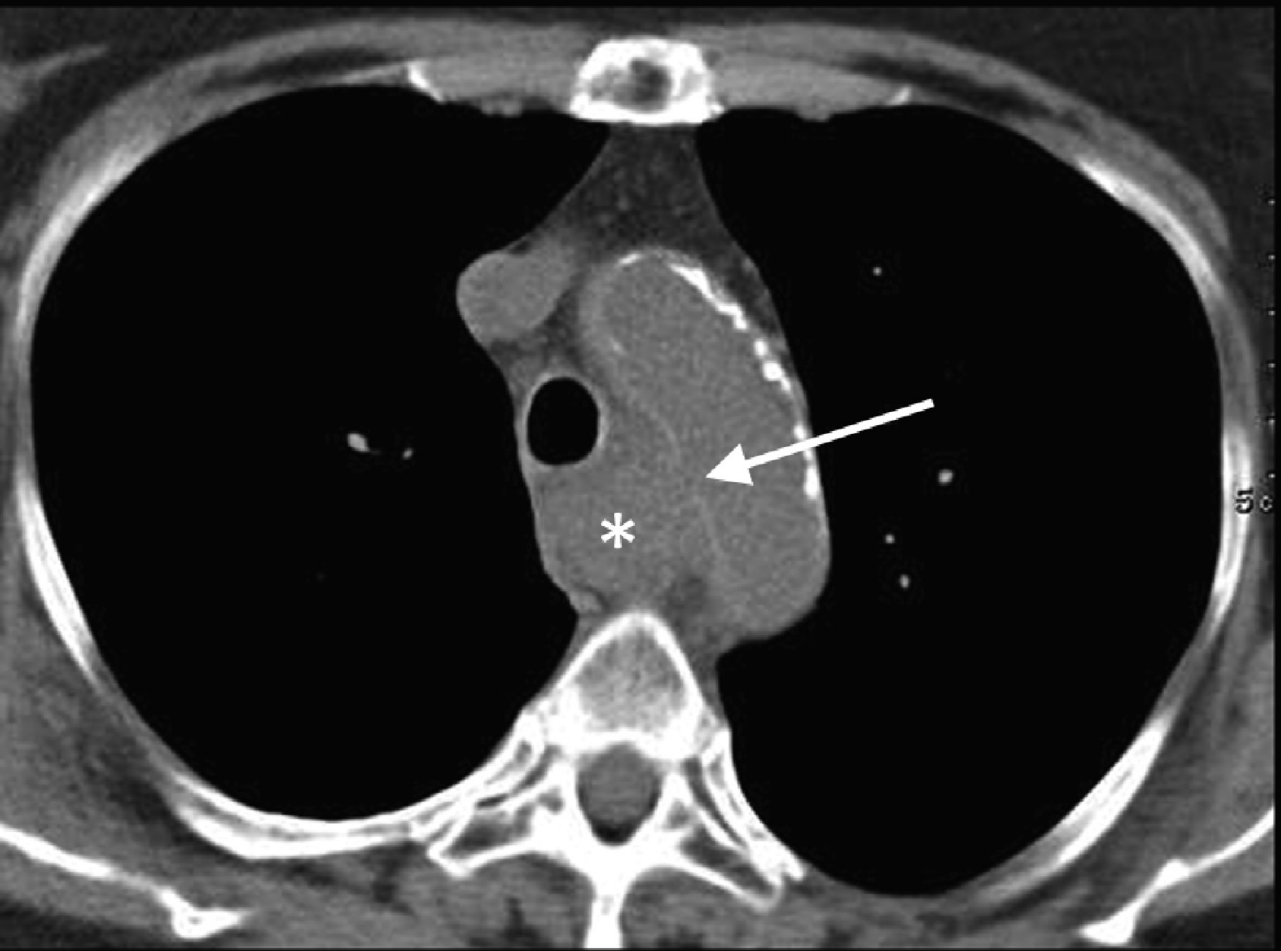
Unenhanced CT showing a retrotracheal, paraortic hyperdense mass (star). There is a small discontinuity in the hyperdense aortic wall, representing the point of fistulation (arrow).

**Figure 2:**
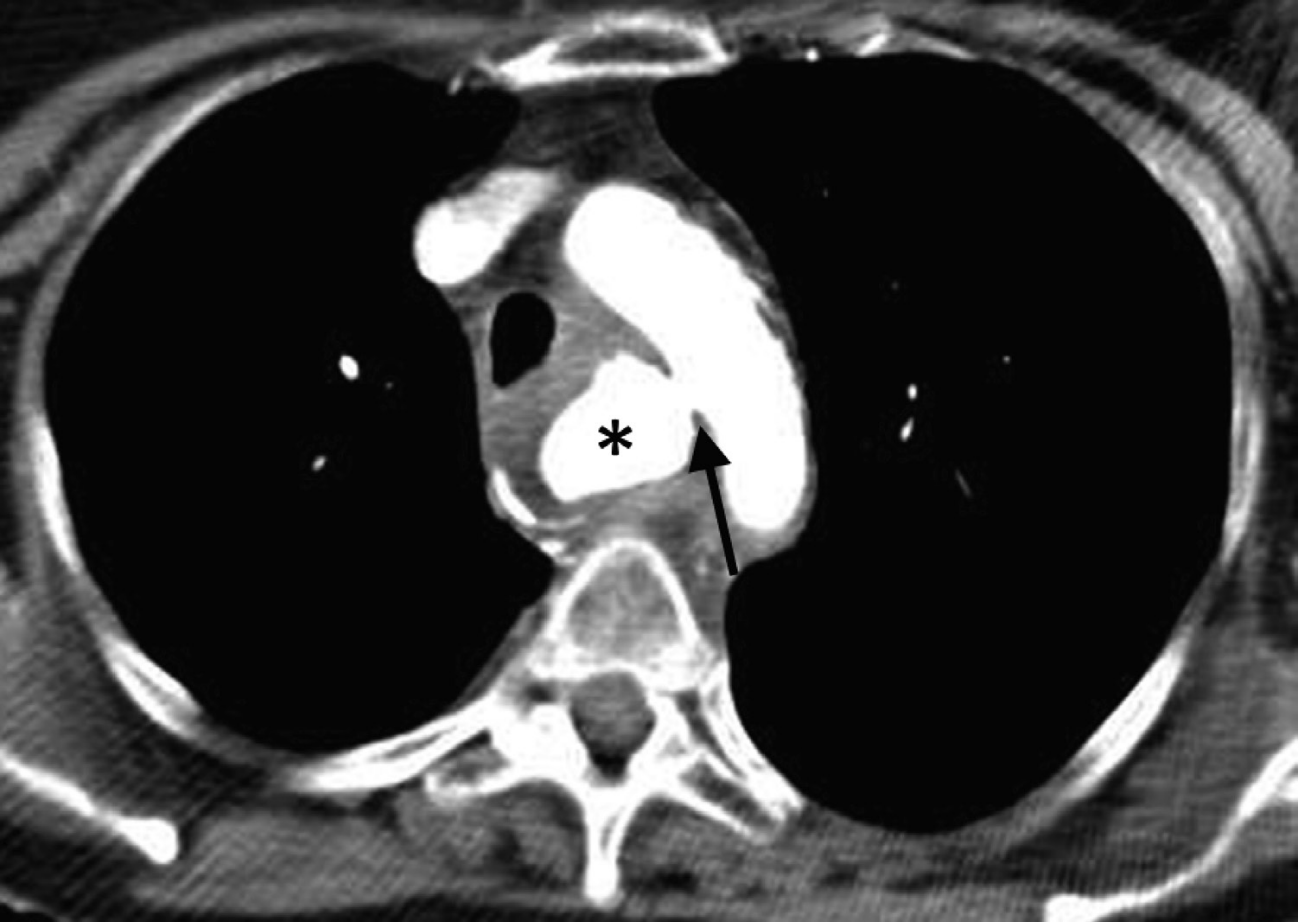
CT angiography showing contrast extravasation into the esophagus (star) from a fistula extending from the medial aspect of the aortic arch (arrow).

**Figure 3:**
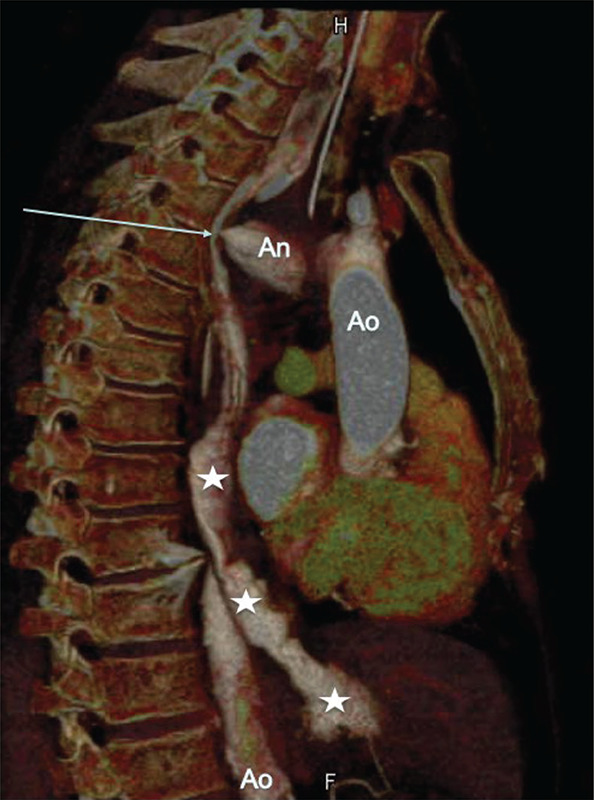
Three‑dimensional (3D) volume‑rendering technique (VRT) image of the CTA in sagittal orientation showing the fistula (arrow) between the aneurysm (An) and contrast material within the esophagus. Aorta (Ao). Esophagus (white stars).

A 76‑year‑old woman was referred for a chest computed tomography (CT) for thoracic pain. She had been treated for esophageal nutcracker syndrome by endoscopic injection of botulinum toxin into the esophageal wall. No procedural complications were reported. An unenhanced CT was performed, showing an esophageal mass that was interpreted as a tumor (Figure 1). The chest pain increased over the following day, with a fresh esophageal hemorrhage observed at an emergency endoscopy. During this endoscopy, a sudden massive hemorrhage occurred. An immediate CT angiography (CTA) of the thoracic aorta was performed, revealing extravasation from the aortic arch into the esophagus, confirming the diagnosis of aorto‑esophageal fistula due to a pseudo‑aneurysm, most likely iatrogenic in nature (Figures 2 and 3). The patient rapidly suffered circulatory collapse due to massive blood loss and expired shortly afterward.

## Discussion

The first description of AEF was in 1818 by Dubrueil; it was caused by the impaction of a chicken bone in the upper esophagus. However, it was only in 1969 that the first patient was successfully treated by surgical intervention [1, 4].

The etiologies of AEF are variable, including ruptured aortic aneurysms, foreign body impaction, post‑procedural complications of intravascular stenting procedures, oncological pathologies, or surgery [1, 3]. With the popularity of interventions and grafts, the incidence of AEF is increasing [3].

Cases of aortic pseudoaneurysm arising from botox injections are exceedingly rare, with three known cases of patients developing inflammatory mediastinitis and pseudoaneurysm, with two of them developing AEF and one perishing as a result [5–7].

The symptoms of AEF include chest pain, dysphagia, and hemorrhage, known as the Chiari triad [1, 3]. Largely aspecific symptoms include gastrointestinal bleeding, back pain, pyrexia, and sepsis [3]. The incidence of gastrointestinal bleeding ranges from 5% to 23% in secondary aortoenteric fistulas [1]. In such cases, the blood loss can be minimal to catastrophic and life‑threatening [1]. The rate of bleeding can be minimal, fluctuating, or cataclysmic [1].

Signs that may be suggestive on chest x‑ray are an enlarged mediastinum [1]. Plain CT may show extraluminal air, mediastinal collections, esophageal thickening, aortic wall dilatation, or rupture, as well as the infiltration of the surrounding fat planes [3]. Contrast‑enhanced CT imaging can show extravasation into the esophagus or an outpouching of contrast [3]. In cases involving the placement of a stent, migration of the stent may be observed [3].

Endoscopy will reveal an esophagus that is either covered with blood clots or is actively bleeding [1].

Therapy is mainly surgery, with techniques ranging from bypass to in‑situ repair using grafts or thoracic endovascular repair (TEVAR), though the optimal treatment is not established [1]. TEVARs have improved the prognosis but are mainly a bridge therapy while awaiting definitive surgical repair, mainly due to prosthetic infection risks [1].

Due to the rarity of AEF, the post‑surgical prognosis is unknown, though some reports suggest a mortality rate of 77% [1, 2]. Untreated cases are always fatal [1].

## Conclusion

AEF is rare, with few described cases occurring after botox injection. As the number of interventions that can cause iatrogenic AEF are increasing, radiological awareness of the entity and its complications is warranted. Prompt diagnosis of AEF is essential, even though the post‑treatment prognosis remains poor.

## References

[r1] Li J, Hu Y, Liu W, Tang J, Zhu S, Zeng C. A successful endovascular aortic repair of aortoesophageal fistula following esophagectomy: A case report and literature review. J Cardiothorac Surg. 2024;19:70. 10.1186/s13019-024-02540-1. 38326831 PMC10848545

[r2] Wong AC, Chou YM, Goh ZNL, Chang KF, Seak CJ. Aortoesophageal fistula—an extremely rare but life‑threatening cardiovascular cause of hematemesis: A case report. Front Cardiovasc Med. 2023;10:1123305. 10.3389/fcvm.2023.1123305. 37153464 PMC10157070

[r3] Gulati A, Kapoor H, Donuru A, Gala K, Parekh M. Aortic fistulas: Pathophysiologic features, imaging findings, and diagnostic pitfalls. Radiographics. 2021;41:1335–1351. 10.1148/rg.2021210004. 34328814

[r4] Yonago RH, Iben AB, Mark JBD. Aortic bypass in the management of aortoesophageal fistula. Ann Thorac Surg. 1969;7(3):235–237. 10.1016/s0003-4975(10)66178-4. 5766744

[r5] Huysmans M, Verbist J, Van den Eynde W, Gryffroy F, Vermeiren K, Cuyle PJ. Treatment of aortoesophageal fistula after endovascular aortic repair for mycotic thoracic aneurysm secondary to endoscopic botulinum toxin injections. Ann Vasc Surg ‑ Brief Rep Innov. 2022;2(1):100046. 10.1016/j.avsurg.2022.100046.

[r6] Chao CY, Raj A, Saad N, Hourigan L, Holtmann G. Esophageal perforation, inflammatory mediastinitis and pseudoaneurysm of the thoracic aorta as potential complications of botulinum toxin injection for achalasia. Dig Endosc. 2015;27:618–621. 10.1111/den.12392. 25329507

[r7] Tan MZY, Whitgift J, Warren H. Mediastinitis, pseudo‑aneurysm formation, aortic bleed, and death from endoscopic botulinum toxin injection. Endoscopy. 2016;48:E186–E187. 10.1055/s-0042-107074. 27213973

